# Impact of an Overnight Stay in the Emergency Department Hallway on Admitted Geriatric Patients

**DOI:** 10.5811/westjem.64046

**Published:** 2026-10-14

**Authors:** Sarah E. Perelman, Melissa A. Meeker, Maura Kennedy, Joshua W. Joseph, Shan W. Liu

**Affiliations:** * Massachusetts General Hospital, Department of Emergency Medicine, Boston, Massachusetts; † Harvard Medical School, Department of Emergency Medicine, Boston, Massachusetts; ‡ Weill Cornell Medicine, Department of Emergency Medicine, New York, New York; § NewYork-Presbyterian Hospital, Department of Emergency Medicine, New York, New York; || Brigham and Women’s Hospital, Department of Emergency Medicine, Boston, Massachusetts

## Abstract

**Introduction:**

Patients frequently board overnight in the emergency department (ED) while awaiting medical admission, sometimes in hallway care spaces. Prior literature suggests that timing of boarding may matter; a single overnight stay in the ED may be associated with increased mortality and adverse events, especially for older adults. Given the deliriogenic ED environment, these effects may be magnified in the hallway. However, little is known about the effects of prolonged hallway stays. We evaluated whether an overnight stay in the ED hallway, compared to an ED room, was associated with increased delirium and adverse outcomes for geriatric patients.

**Methods:**

We conducted a retrospective multisite cohort database study of patients aged 65 years and older admitted to a general medicine service January 1, 2022, to December 31, 2023, who spent an entire night (midnight to 8 am) in the ED prior to admission. We compared those who spent the entire night in the ED hallway with those who were in ED rooms. Our primary outcome was a composite outcome for incident delirium and severe agitation (defined by the I*nternational Classification of Diseases*, 10^th^ Rev, code for delirium, positive nursing screen, use of parenteral antipsychotics, and/or physical restraints, since delirium was not routinely screened for at these sites). Secondary outcomes included hospital length of stay (LOS), inpatient and 30-day mortality, and 30-day ED revisit and readmission. We implemented mixed-effect regression models and adjusted for confounders.

**Results:**

A total of 8,576 patients met inclusion criteria, including 1,231 patients (14%) in the hallway group. Overall, 847 patients (9.9%) developed incident delirium and severe agitation: 160 (13%) of the hallway group and 687 (9%) of the non-hallway group. An overnight stay in the ED hallway was not associated with delirium and severe agitation (OR, 1.06, 95% CI, 0.58–1.94; *P* = .84), but increased ED LOS was independently associated with delirium and severe agitation (OR, 1.01; 95% CI, 1.0–1.01 per hour; *P* = .01). There was no association between an overnight stay in the hallway and secondary outcomes except increased hospital LOS (OR 1.01, 95% CI, 1.00–1.01 per hour; *P* = .01).

**Conclusion:**

An overnight in the ED hallway care space was not associated with increased delirium and severe agitation for geriatric patients, but prolonged ED LOS was associated with this outcome. Hospital systems should consider prioritizing geriatric patients for expedited transfer from the ED to inpatient units, and prospective research with consistent delirium screening is needed to rigorously evaluate the safety of hallway care for older adults.

## INTRODUCTION

Admitted patients frequently experience prolonged emergency department (ED) length of stay (LOS) prior to transfer to inpatient units due to the boarding and crowding crisis, despite emerging evidence that spending extended time in the ED is associated with delirium, increased hospital LOS, and mortality.^1^–^3^ These negative outcomes are especially pronounced for older adults.^2^,^3^ As EDs struggle with capacity challenges related to boarding, using ED hallway care spaces has increased. Few studies have evaluated the safety of hallway care. Our recent paper suggested that extended ED hallway care may be associated with delirium for older persons living with dementia.^4^

Importantly, emerging evidence suggests that the timing of ED exposure may also matter. A multicenter cohort study found that even a single overnight stay in the ED while awaiting admission was associated with and adverse events.^2^ Determining whether an overnight ED hallway stay is associated with delirium could inform whether prioritizing standard treatment rooms for high-risk boarders serves as a feasible prevention strategy when immediate transfer to inpatient units is impossible. We conducted a multisite retrospective cohort study to evaluate the association between an overnight ED hallway stay and incident delirium and severe agitation. This study is a secondary analysis of our prior investigation into the impacts of prolonged hallway care.^4^ We hypothesized that an overnight hallway stay would be associated with delirium and severe agitation, and that the risk would be worse in persons living with dementia.

## METHODS

We performed a retrospective cohort database study of older adults (at least 65 years of age) who presented to the ED and were subsequently admitted to a general medicine service at two urban, quaternary care EDs in the United States between January 1, 2022, and December 31, 2023, using the electronic health record (EHR). We used aggregated data to allow for a large sample size and followed the Strengthening the Reporting of Observational Studies in Epidemiology (STROBE) guidelines.^5^ We applied the same exclusion criteria as in our prior work. We excluded patients with ED LOS fewer than 8 hours and who had delirium on arrival or a psychiatric diagnosis, and we only included each patient’s most recent visit.^4^ For this study, we additionally excluded ED encounters in which the patient was not in the ED for a full night, defined as from midnight to 8 am. Although “sundowning”—the exacerbation of confusion or delirium later in the day—can occur prior to midnight, we chose this window following the Roussel et al definition of “overnight” associated with increased mortality.^2^

Eligible participants were dichotomized into either the the hallway group (those who spent an entire overnight period from midnight to 8 am in the ED hallway) or the non-hallway group (those who spent the overnight period in the ED but not in hallway care space). Non-hallway care spaces included both primarily private rooms and curtained (semi-private) rooms. Given brief moves into the hallway from rooms for sensitive exams or when resuscitations or other critical patients arrive sometimes occurred, we did not exclude patients who were moved from a room to hallway for part of the night from the non-hallway group.

**Population Health Research Capsule**

What do we already know about this issue?

*Prolonged emergency department (ED) length of stay is associated with delirium for older adults admitted to the hospital.*

What was the research question?

*Is spending an overnight in the ED hallway associated with increased delirium and severe agitation for admitted geriatric patients?*

What was the major finding of the study?

*An overnight in an ED hallway vs ED room was not associated with increased delirium and severe agitation (OR 1.06; 95% CI, 0.58–1.94] P = .84).*

How does this improve population health?

*Prioritizing transfer of admitted older adults to an inpatient unit remains the priority for mitigating delirium associated with a prolonged ED length of stay.*

The primary outcome was incident delirium and severe agitation, defined as a composite outcome of *International Classification of Diseases*, 10^th^ Rev, (*ICD-10*) code for delirium, positive nursing screen, use of parenteral antipsychotics, and/or physical restraints, as previously described.^3^,^4^ Both institutions started educational initiatives for physicians and nurses aimed at identifying delirium and a Confusion Assessment Method (CAM) score was integrated into the medical record, but delirium screening was not consistently performed. Secondary outcomes included hospital LOS in days, inpatient and 30-day mortality, and 30-day ED revisit and readmission. We also examined whether there was an interaction between each of these outcomes and a history of dementia.

We employed structured query language to abstract encounter and patient data from our EHR (Epic Systems Corporation), including potential confounders determined a priori (age, ED LOS, history of dementia or delirium, acuity as measured by Emergency Severity Index [ESI] level], Charlson Comorbidity Index [CCI], sex, insurance carrier, and combined race and ethnicity). First, we examined our outcomes with descriptive statistics. Next, we evaluated bivariate associations between hallway exposure and each patient demographic and visit characteristic with a chi-squared test for categorical variables and a Kruskal-Wallis test for continuous variables. We modeled the association between our composite outcome and hallway care using mixed-effect logistic regression, which included fixed effects to adjust for confounders (as noted above), a random effect to control for site-level correlation, and an interaction term to evaluate whether a prior diagnosis of dementia mediated the association. We employed the same methods for each of the secondary outcomes, using logistic regression for each binary outcome and Poisson regression for LOS.

We repeated the analysis for primary and secondary outcomes with overnight hallway exposure as a continuous variable as a sensitivity analysis.

## RESULTS

Of the 11,105 patients included in the original study, 8,576 (77.2%) spent an overnight in the ED and were included in this study, and 2,529 participants were excluded for not being in the ED for a complete overnight stay. The median age was 77 years (interquartile range [IQR], 71–84), and the median ED LOS was 30 hours (IQR, 24–45); most participants were non-Hispanic White (Table 1). The hallway group included 1,231 patients (14%), and the non-hallway group included 7,345 patients (86%). No significant differences were observed in race, acuity, ED LOS, or history of dementia (all *P* > .05); however, a larger proportion of men spent the overnight in a hallway care space (52% versus 49%; *P* = .02).

Overall, 847 patients (9.9%) developed incident delirium and severe agitation: 160 patients (13%) of the hallway group and 687 patients (9%) of the non-hallway group. Of these patients, 248 (29%) had a positive CAM screen, 257 (30%) received parenteral antipsychotics, 39 (5%) had delirium diagnosis, and 564 (67%) (n=564) had restraints placed. Median hospital LOS was 5 days in both groups (IQR, 2–9] in the hallway group and [IQR, 3–9] in the non-hallway group; *P* = .07). Overall, in-hospital mortality was 4% (378) with 3% (43) of the hallway group and 4% (335) of the non-hallway group (*P* = .11). The 30-day return rate was 13% (1,128), including 13% (160) of the hallway group and 13% (968) of the non-hallway group (*P* =.90). Of those who returned, 30-day readmission was 40% (446), consisting of 35% (56) of the hallway group and 40% (390) of the non-hallway group (*P* = .14).

Our multivariable regression revealed no association between an overnight in the hallway and the development of delirium and severe agitation (OR, 1.06; 95% C, 0.58–1.94; *P* =.84) (Figure) when controlling for covariates (Table 2). Furthermore, there was no interaction between overnight hallway LOS and history of dementia, and development of delirium/severe agitation (Table 2). The ED LOS was associated with delirium and severe agitation (OR, 1.01 [1.00–1.01; *P* =.01).

An overnight in the hallway was associated with longer hospital LOS when controlling for covariates (OR, 1.09; 95% CI, 1.07–1.12; *P* < .001). The interaction between a history of dementia and hallway care was associated with a decrease in hospital LOS (relative risk, 0.90; 0.85–0.96; *P* < .001). There was no significant association between hallway care and ED revisit (OR, 0.97; 0.80–1.18; *P* = .78), readmission (OR, 0.70; 0.47–1.05; *P* = .08), 30-day mortality (OR 0.87; 0.60–1.28; *P* = .48), or inpatient mortality (OR, 0.35; 0.03–3.83; *P* = .39), nor was a significant interaction between hallway care and dementia observed for any of these secondary outcomes.

Our sensitivity analysis with hallway time as a continuous variable also revealed an association between hallway care and longer hospital LOS (OR, 1.01; 1.00–1.01; *P* <.001) (Table S1). There was no association between hallway care and delirium and severe agitation or other secondary outcomes. There was no interaction observed between hallway care and a history of dementia for any of the outcomes.

## DISCUSSION

Our work fails to support overnight room prioritization as a delirium prevention measure for admitted older adults boarding in the ED, instead suggesting that ED LOS regardless of location within the ED may be associated with delirium. Given that Roussel et al reported a single overnight in the ED was associated with increased hospital LOS and mortality for geriatric patients,^2^ we hypothesized that these effects may be mediated by delirium and magnified in the hallway. In our cohort, 9.9% developed delirium or severe agitation; however, similar to the findings of Elder et al,^1^ ED LOS rather than hallway placement was associated with delirium. A relatively small number of patients (160) were positive for delirium in the hallway group, raising the possibility that the study was underpowered to detect a =difference in groups, especially as there was a trend toward as association between hallway care and delirium that did not meet significance (OR, 1.06, 95% CI, 0.58–1.94; *P* = .84). However, a post-hoc power calculation was high (.98).

This research builds on a growing body of literature suggesting that prolonged ED LOS is harmful, with a recent study noting that each hour in the ED is associated with a 2% increased risk of delirium or severe agitation.^4^ Our effect size was not larger than those in prior studies, suggesting that timing at night may not be a driving factor, but rather duration of ED LOS as a whole regardless of night or day. This emerging body of evidence highlights the importance of minimizing boarding time for geriatric patients, as proposed by the recent Centers for Medicare & Medicaid Services (CMS) Age-Friendly Hospital Measure.^6^

Our work also highlights key risk factors for incident delirium and severe agitation after an overnight in the ED: a history of dementia, prior delirium episodes, older age, and higher acuity. This supports prior literature on delirium risk factors^11^; these results could also be actionable for hospital systems when deciding which patients might be better served by prioritization for an inpatient room when beds are scarce.

Our lack of observed difference in incident delirium and severe agitation between groups may be partially explained because many factors in the ED environment thought to be associated with delirium (such as night-time clinical tests and fragmented sleep from noise) may be similar between traditional rooms and hallway treatment space, particularly as some rooms are curtained or semi-private. It is also possible that differences in patient factors between the groups placed in the hallway and rooms may account for the lack of impact of hallway care we observed. These differences may not be fully accounted for by ESI level and CCI, because when a clinician decides where to room a patient, they are likely using other clinical measures of stability based on their assessment and experience that our methods would not capture. Prior work suggests triage nurses place lower acuity patients in the hallways.^7^ Supporting this, the finding that persons living with. dementia in the hallway group have a shorter hospital LOS seems counterintuitive clinically. It is possible that features of their presentation or chief complaint are deemed low risk by clinicians, leading to hallway placement, which are not fully adjusted for by ESI level and CCI. It is also possible that family members prefer that persons living with dementia remain at home due to concerns regarding given fear of hospital-induced delirium, a condition for which this population carries a significantly higher risk.^11^

Interestingly, hallway care was associated with a longer overall hospital LOS but not with delirium, severe agitation, or mortality. This finding may indicate that patients who spend the overnight period in a hallway serve as a marker of severe hospital crowding, which exerts widespread systemic strain and causes care delays for all patients.^12^ As the hallway group is a smaller cohort, it is possible overnight in the hallway only occurred at times of most severe crowding. However, it is also possible that the care was different or not progressing as quickly for these patients. Qualitative work has suggested that nurses struggle more to mobilize hallway patients and provide personalized care^13^ and that ack of mobility can lead to increased LOS.^14^ Additionally, our delirium and severe agitation outcome was measured by proxy; consequently, hospital LOS may capture some vulnerable patients who were missed by this proxy metric. Furthermore, some lower acuity patients may remain hospitalized due to social factors, such as awaiting nursing home placement. This specific population frequently experiences prolonged hospital LOS while social work services coordinate complex disposition plans.^15^

## LIMITATIONS

The lack of association we observed between night-time care in the ED hallway treatment area and delirium or severe agitation may also be due to the significant limitations in our methods. Delirium is under-recognized in the ED and rarely documented in the medical record; only a third of cases may be identified and documented by emergency physicians.^8^ Acknowledging the lack of ability to detect delirium by diagnosis code alone, we included the use of parenteral antipsychotics and physical restraints in our composite outcome as a proxy for hyperactive or mixed delirium. However, our outcome criteria may still miss hypoactive delirium, which may account for up to 90% of all delirium cases^9^ and carries a higher mortality than hyperactive delirium.^10^ Only 9.9% of our cohort developed delirium, a rate lower than previously reported incidences among hospitalized older adults.^11^ This discrepancy suggests that our methods underestimated the true incidence, thereby biasing our findings toward the null hypothesis even if a true difference exists between overnight ED room and hallway care.

Differences in delirium detection may also exist across groups and create a systematic bias; on the one hand, hyperactive delirium may be better identified in hallways because patients are more visible. On the other hand, more consistent screens may be done for patients assigned to rooms. Additionally, crossover contamination may have occurred because we did not exclude patients from the non-hallway group if they spent part of the night in the hallway, although this choice was made to reflect the dynamic nature of ED room assignment given the possibility of brief moves of patients from rooms to a hallway when resuscitations arrived or to allow for sensitive exams or procedures to be performed for patients with a primary room assignment in the hallway. Because only 13% (984 patients) of the non-hallway group experienced such moves, we think the impact would not significantly change the results. Finally, we specifically examined delirium or severe agitation after an overnight stay in the ED, but some patients may develop delirium in a shorter timeframe; it is unknown whether a short hallway stay is associated with delirium and severe agitation, which could be a topic for future research, as proportion of time in the hallway may be associated with delirium.^16^

This underscores the importance of future prospective work, ideally with multicenter collaborations to achieve adequate power (such as through the Geriatric Emergency Applied Research Network),^17^ with consistent screening using validated tools to assess for delirium to determine whether there is a true risk of overnight hallway care.

## CONCLUSION

Our retrospective study found that an overnight stay in the ED hallway versus ED room was not associated with our composite outcome for delirium and severe agitation; however, it was associated with longer hospital length of stay. We also observed an association between ED LOS and delirium or severe agitation. As hospitals create policies to follow the CMS Age-Friendly Hospital Measure, our study supports that minimizing ED boarding may prevent delirium. Future prospective research in which validated tools are used consistently to screen for delirium is needed to determine whether certain patients are at risk of developing delirium from ED hallway care.

## Supplementary Information



## Figures and Tables

**Figure:**
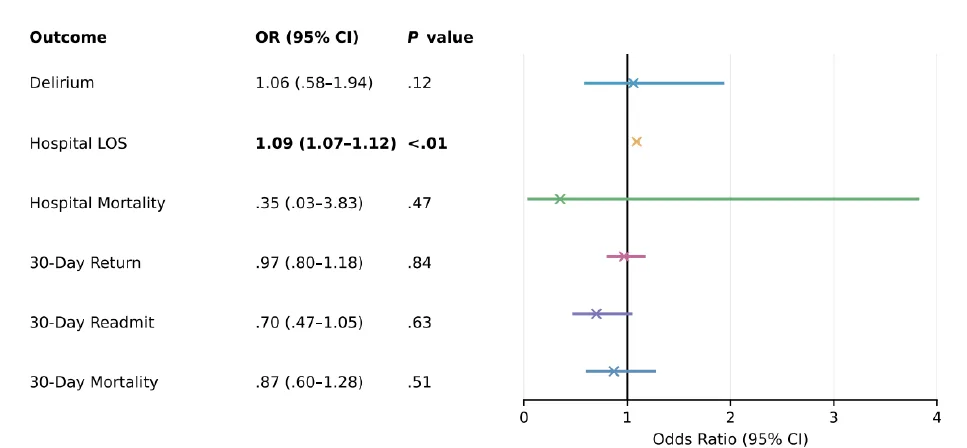
Odds of delirium, severe agitation, and secondary outcomes by adjusted generalized linear mixed-effect regression models stratified by overnight hallway placement in a retrospective cohort study of older adults admitted to general medicine with > 8-hour emergency department length of stay. Poisson regression was applied to Hospital LOS (with rate ratio as outcome) and logistic regression was applied for all other outcomes (with odds ratio as outcome). Figure generated using ChatGPT (GPT-4o), reviewed by the authors for accuracy.

**Table 1:** Demographics and descriptive analysis of outcomes of delirium, hospital length of stay (LOS), readmission, and mortality in a retrospective cohort study of older adults admitted to general medicine with > 8-hour emergency department LOS.

		Overall (N = 8,576)	Overnight hallway (n = 1,231)	Overnight non-hallway (n = 7,345)	P value
Age (years)^1^		77 (71–84)	77 (70–83)	77 (71–84)	.001
Sex	Male	4,207 (49%)	643 (52%)	3,564 (49%)	.02
	Female	4,369 (51%)	588 (48%)	3,781 (51%)	
Combined Race and Ethnicity	Hispanic	664 (8%)	103 (8%)	561 (8%)	.43
	Non-Hispanic Black	742 (9%)	96 (8%)	646 (9%)	
	Non-Hispanic White	6,184 (72%)	877 (71%)	5,307 (72%)	
	Non-Hispanic Other	597 (7%)	91 (7%)	506 (7%)	
	Unknown	389 (5%)	64 (5%)	325 (4%)	
Site	Site A	1,867 (22%)	109 ( 9%)	1,758 (24%)	< .001
	Site B	6,709 (78%)	1,122 (91%)	5,587 (76%)	
Charlson Comorbidity Index^1^		2 (0, 4)	2 (0, 3)	2 (0, 4)	< .001
History of dementia		1,019 (12%)	156 (13%)	863 (12%)	.38
Insurance	Private	560 (7%)	75 (6%)	485 (7%)	.02
	Public	7,838 (91%)	1,118 (91%)	6,720 (91%)	
	Other	178 (2%)	38 (3%)	140 (2%)	
Acuity	1 to 2	4,441 (52%)	620 (50%)	3,821 (52%)	.55
	3 to 5	4,063 (47%)	601 (49%)	3,462 (47%)	
	Missing	72 (1%)	10 (1%)	62 (1%)	
ED length of stay (hours)^1^		30 (24, 45)	30 (24, 45)	30 (24, 44)	.26
Delirium		847 (9.9%)	160 (13%)	687 ( 9%)	< .001
Hospital length of stay (days)^1^		5 (3, 9)	5 (2, 9)	5 (3, 9)	.07
Hospital mortality		378 (4%)	43 (3%)	335 (5%)	.11
30-day return		1,128 (13%)	160 (13%)	968 (13%)	.90
30-day readmit^2^		446 (40%)	56 (35%)	390 (40%)	.14
30-day mortality		681 (8%)	75 (6%)	606 (8%)	.01

1 Presented as median (IQR).

2 Among those with 30-day return.

*ED*, emergency department.

**Table 2:** Adjusted generalized linear mixed-effect regression models of delirium, severe agitation and secondary outcomes by overnight hallway exposure in a retrospective cohort study of older adults admitted to general medicine with >8 hour emergency department length of stay.

	Delirium Odds Ratio [95% CI]	Hospital LOS Rate Ratio [95% CI]	Hospital Mortality Odds Ratio [95% CI]	30-Day Return Odds Ratio [95% CI]	30-Day Readmit Odds Ratio [95% CI]	30-Day Mortality Odds Ratio [95% CI]
Hallway Group – Adjusted	1.06 [0.58, 1.94]	**1.09** [1.07, 1.12]	0.35 [0.03, 3.83]	0.97 [0.80, 1.18]	0.70 [0.47, 1.05]	0.87 [0.60, 1.28]
History of Dementia	**2.33** [1.86, 2.92]	**1.17** [1.14, 1.20]	1.02 [0.73, 1.44]	1.12 [0.89, 1.41]	1.18 [0.77, 1.81]	1.14 [0.89, 1.48]
Interaction between Hallway Patient and History of Dementia	1.18 [0.75, 1.85]	**0.90** [0.85, 0.96]	0.58 [0.21, 1.56]	1.01 [0.60, 1.69]	2.40 [0.90, 6.40]	0.71 [0.36, 1.42]
History of Delirium	**2.37** [1.83, 3.07]	**1.20** [1.16, 1.24]	1.47 [0.97, 2.24]	1.15 [0.86, 1.53]	1.17 [0.68, 2.00]	**1.62** [1.19, 2.22]
ED Length of Stay, hours	**1.01** [1.00, 1.01]	**1.004** [1.00, 1.00]	**0.99** [0.99, 1.00]	**1.004** [1.00, 1.01]	1.00 [0.99, 1.01]	**0.99** [0.99, 1.00]
Age at Arrival, years	**1.03** [1.02, 1.04]	**0.995** [0.99, 1.00]	**1.06** [1.04, 1.07]	**0.98** [0.97, 0.99]	**1.02** [1.00, 1.03]	**1.05** [1.04, 1.06]
Female Sex (compared to male)	**0.77** [0.67, 0.89]	**0.94** [0.93, 0.95]	0.94 [0.76, 1.16]	**0.88** [0.77, 1.00]	1.08 [0.84, 1.38]	**0.81** [0.69, 0.95]
Charlson Comorbidity Index	**0.95** [0.91, 0.98]	**1.02** [1.01, 1.02]	**1.15** [1.09, 1.20]	**1.07** [1.03, 1.10]	1.04 [0.98, 1.11]	**1.15** [1.11, 1.19]
Insurance (compared to private)						
Public	0.95 [0.69, 1.30]	**1.04** [1.00, 1.07]	1.16 [0.69, 1.95]	**1.63** [1.21, 2.19]	1.24 [0.68, 2.26]	1.06 [0.73, 1.54]
Other	1.26 [0.72, 2.19]	1.64 [1.56, 1.72]	1.16 [0.44, 3.08]	1.68 [0.99, 2.83]	1.31 [0.45, 3.77]	0.90 [0.41, 1.95]
Race and Ethnicity (compared to non-Hispanic White)						
Hispanic	0.80 [0.59, 1.09]	**0.87** [0.85, 0.90]	0.65 [0.40, 1.08]	1.23 [0.99, 1.54]	0.73 [0.47, 1.13]	**0.60** [0.41, 0.88]
Non-Hispanic Black	1.09 [0.83, 1.42]	**1.13** [1.10, 1.15]	0.87 [0.56, 1.35]	1.18 [0.95, 1.46]	**0.48** [0.30, 0.75]	0.80 [0.57, 1.10]
Non-Hispanic Other	0.93 [0.69, 1.24]	**0.92** [0.89, 0.95]	1.09 [0.73, 1.61]	1.07 [0.83, 1.37]	1.25 [0.78, 2.00]	0.95 [0.69, 1.31]
Unknown	1.35 [0.99, 1.84]	1.00 [0.97, 1.04]	1.14 [0.71, 1.83]	**0.57** [0.38, 0.83]	1.09 [0.51, 2.34]	0.86 [0.57, 1.29]
Acuity (compared to 1 to 2)						
3 to 5	**0.72** [0.62, 0.84]	**1.02** [1.00, 1.03]	**0.59** [0.47, 0.73]	1.12 [0.98, 1.27]	**0.74** [0.58, 0.95]	**0.72** [0.61, 0.85]
Missing	1.77 [0.96, 3.26]	1.06 [0.98, 1.15]	0.45 [0.11, 1.87]	0.50 [0.20, 1.25]	0.82 [0.13, 5.20]	0.99 [0.44, 2.23]

Bold = statistically significant to *P* < .05.

Values are shown to 2 decimal places except where additional precision is required to indicate statistical significance

Poisson regression was applied to hospital LOS and logistic regression was applied for all other outcomes.

*LOS*, length of stay.
