# Supplementary material for: Impact of an Overnight Stay in the Emergency Department Hallway on Admitted Geriatric Patients
Source: West J Emerg Med. 2026 Oct 14;27(6):1751–7. doi: 10.5811/westjem.64046 (PMC13761714; doi:10.5811/westjem.64046)
Supplement: Supplementary file 1 [file wjem-27-1751-s001.pdf]

Table S1. Adjusted Generalized Linear Mixed-Effect Regression Models of Delirium and Secondary Outcomes with Overnight Hallway Time as a Continuous Variable in a Retrospective Cohort Study of Older Adults Admitted to General Medicine with >8 Hour ED Stays

	**Delirium Odds Ratio [95% CI]**	**Hospital LOS Rate Ratio [95% CI]**	**Hospital Mortality Odds Ratio [95% CI]**	**30-Day Return Odds Ratio [95% CI]**	**30-Day Readmit Odds Ratio [95% CI]**	**30-Day Mortality Odds Ratio [95% CI]**
Time in hallway care space (hours)	1.03 [0.99, 1.07]	**1.01 [1.00, 1.01]**	0.97 [0.90, 1.05]	1.00 [0.98, 1.02]	0.99 [0.96, 1.03]	0.99 [0.95, 1.03]
History of Dementia	**2.36 [1.88, 2.97]**	**1.14 [1.11, 1.18]**	1.05 [0.75, 1.49]	1.10 [0.87, 1.40]	1.16 [0.75, 1.80]	1.17 [0.90, 1.51]
Interaction between Time in Hallway and History of Dementia	1.01 [0.97, 1.05]	1.00 [1.00, 1.01]	0.92 [0.82, 1.03]	1.00 [0.96, 1.05]	1.07 [0.98, 1.18]	0.96 [0.89, 1.03]
History of Delirium	**2.37 [1.83, 3.07]**	**1.20 [1.16, 1.24]**	1.47 [0.97, 2.24]	1.15 [0.86, 1.54]	1.19 [0.69, 2.03]	**1.62 [1.18, 2.22]**
ED Length of Stay, hours	**1.00 [1.00, 1.01]**	**1.00 [1.00, 1.00]**	**0.99 [0.99, 1.00]**	**1.004 [1.00, 1.01]**	1.00 [0.99, 1.01]	**0.99 [0.99, 1.00]**
Age at Arrival, years	**1.03 [1.02, 1.04]**	**0.99 [0.99, 1.00]**	**1.05 [1.04, 1.07]**	**0.98 [0.97, 0.99]**	**1.02 [1.00, 1.03]**	**1.05 [1.04, 1.06]**
Female Sex (compared to male)	**0.77 [0.67, 0.89]**	**0.94 [0.93, 0.95]**	0.94 [0.76, 1.16]	0.88 [0.78, 1.00]	1.08 [0.85, 1.39]	**0.81 [0.69, 0.95]**
Charlson Comorbidity Index	**0.95 [0.91, 0.98]**	**1.02 [1.01, 1.02]**	**1.15 [1.09, 1.20]**	**1.07 [1.03, 1.10]**	1.05 [0.99, 1.11]	**1.15 [1.11, 1.19]**
Insurance (compared to private)						
Public	0.95 [0.69, 1.30]	**1.03 [1.00, 1.07]**	1.16 [0.69, 1.95]	**1.62 [1.21, 2.19]**	1.22 [0.67, 2.23]	1.06 [0.73, 1.54]
Other	1.27 [0.73, 2.21]	**1.64 [1.56, 1.73]**	1.16 [0.44, 3.07]	1.67 [0.99, 2.82]	1.22 [0.42, 3.49]	0.89 [0.41, 1.93]
Race and Ethnicity (compared to non-Hispanic White)						
Hispanic	0.79 [0.58, 1.08]	**0.87 [0.85, 0.90]**	0.65 [0.40, 1.08]	1.23 [0.99, 1.54]	0.75 [0.48, 1.16]]	**0.61 [0.42, 0.89]**
Non-Hispan	1.08 [0.	**1.12 [1.10,**	0.86 [0.55,	1.18 [0.95,	**0.49 [0.31,**	0.80 [0.57,

ic	83, 1.42]	**1.15]**	1.34]	1.46]	**0.76]**	1.11]
Black Non-Hispanic	0.93 [0.69, 1.24]	**0.92 [0.90, 0.95]**	1.09 [0.73, 1.62]	1.07 [0.83, 1.37]	1.26 [0.79, 2.02]	0.95 [0.69, 1.31]
Other	1.35 [0.99, 1.85]	1.00 [0.97, 1.04]	1.14 [0.71, 1.83]	**0.57 [0.38, 0.83]**	1.09 [0.51, 2.34]	0.86 [0.57, 1.29]
Unknown						
Acuity (compared to 1 to 2)						
3 to 5	**0.72 [0.62, 0.83]**	1.01 [1.00, 1.03]	**0.59 [0.47, 0.73]**	1.12 [0.98, 1.27]	**0.74 [0.58, 0.95]**	**0.72 [0.61, 0.85]**
Missing	1.75 [0.95, 3.23]	1.06 [0.98, 1.15]	0.46 [0.11, 1.89]	0.50 [0.20, 1.26]	0.87 [0.14, 5.53]	1.00 [0.45, 2.24]

Bold = statistically significant to p<0.05

Values are shown to 2 decimal places except where additional precision is required to indicate statistical significance

Poisson Regression was applied to Hospital LOS and Logistic Regression was applied for all other outcomes.
